# Biomimetic superhydrophobic metal/nonmetal surface manufactured by etching methods: A mini review

**DOI:** 10.3389/fbioe.2022.958095

**Published:** 2022-08-05

**Authors:** Shangjie Ge-Zhang, Hong Yang, Haiming Ni, Hongbo Mu, Mingming Zhang

**Affiliations:** ^1^ College of Science, Northeast Forestry University, Harbin, China; ^2^ College of Bioinformatics Science and Technology, Harbin Medical University, Harbin, China

**Keywords:** bionic modification, superhydrophobic surface, micro/nanostructure, etching modification method, chemical etching method, laser-etching method

## Abstract

As an emerging fringe science, bionics integrates the understanding of nature, imitation of nature, and surpassing nature in one aspect, and it organically combines the synergistic complementarity of function and structure–function integrated materials which is of great scientific interest. By imitating the microstructure of a natural biological surface, the bionic superhydrophobic surface prepared by human beings has the properties of self-cleaning, anti-icing, water collection, anti-corrosion and oil–water separation, and the preparation research methods are increasing. The preparation methods of superhydrophobic surface include vapor deposition, etching modification, sol–gel, template, electrostatic spinning, and electrostatic spraying, which can be applied to fields such as medical care, military industry, ship industry, and textile. The etching modification method can directly modify the substrate, so there is no need to worry about the adhesion between the coating and the substrate. The most obvious advantage of this method is that the obtained superhydrophobic surface is integrated with the substrate and has good stability and corrosion resistance. In this article, the different preparation methods of bionic superhydrophobic materials were summarized, especially the etching modification methods, we discussed the detailed classification, advantages, and disadvantages of these methods, and the future development direction of the field was prospected.

## 1 Introduction

Nature is always the source of our inspiration. By observing the structural diversity, functional specificity, and environmental responsiveness of natural organisms, human beings have discovered many different biological structures and functions, thus creating more and more new materials and structures through technological innovation and cross-fertilization across disciplines. Bionic materials are materials prepared by simulating the unique structure or characteristics of organisms (Suresh Kumar et al., 2020; Koch et al., 2008). Biomimetic superhydrophobic surfaces, which are prepared by using the superhydrophobic phenomena related to biological structures in nature (Table 1), such as lotus leaves (Sun and Guo, 2019; Lv et al., 2020), butterfly wings (Zheng et al., 2007; Shao et al., 2019) and rice leaves (Wu et al., 2011; Lee et al., 2013; Rius-Ayra et al., 2018), have attracted wide attention and research because of outstanding self-cleaning (Barthlott and Neinhuis, 1997; Fürstner et al., 2005; Ming et al., 2005), anti-icing (Liu et al., 2020; Sun et al., 2020), anti-corrosion (Liu et al., 2014; Wei et al., 2021a; Zhang et al., 2022), and oil–water separation (Wang et al., 2015a; Saleh et al., 2020; Rasouli et al., 2021; Yao et al., 2021) properties. In recent years, with the deepening of the research in the micro-field, micro-nano materials have developed rapidly, and they have been developed into intelligent responsiveness (Li et al., 2017a; Chang et al., 2018; Li et al., 2021a), environmental remediation (Kumari et al., 2019; Sajjadi et al., 2021), biodegradability (Li et al., 2021b; Li et al., 2022a; Li et al., 2022b), nano-probe imaging (Li et al., 2016; Li et al., 2017b; Li et al., 2019a; Li et al., 2022c), and other characteristics, which are widely used in many fields (Waked, 2011; Khandelwal et al., 2016; Scalisi, 2017; Li et al., 2019b; Siddiqui et al., 2019), especially medicine (Li et al., 2021c; Lu et al., 2021). Biomimetic superhydrophobic surface combines the cutting-edge technologies of bionics and micro-nano fields, and has great development prospects.

**TABLE 1:** The surface structure of typical organisms.

Biological surface	Properties	References
Lotus leaf	Superhydrophobic, self-cleaning	Barthlott and Neinhuis, (1997)
Rose petal	Superhydrophobic, high surface adhesion	(Feng et al., 2008; Zheng et al., 2019)
Rice leaf	Superhydrophobic, directional transport	(Feng et al., 2002; Wu et al., 2011)
Nepenthes	Directional transport, water harvesting	(Bohn and Federle, 2004; Wong et al., 2011; Chen et al., 2016)
Purple setcreasea	Double-sided superhydrophobic	Guo and Liu, (2007)
Watermelon leaf	Single-order scale hydrophobic structure	Guo and Liu, (2007)
Peanut leaf	Superhydrophobic, high surface adhesion	(Yang et al., 2014; Gou and Guo, 2018; Qu et al., 2020)
Bamboo leaf	Anti-icing, high surface adhesion	(Yuan et al., 2014; Wan et al., 2021; Wan et al., 2022)
Gecko foot	High surface adhesion, self-cleaning	(Autumn et al., 2002; Wang et al., 2012; Watson et al., 2015; Basak, 2020)
Cicada wing	Self-cleaning, anti-reflective	(Watson and Watson, 2004; Stoddart et al., 2006; Zhang et al., 2006; Xie et al., 2017; Román‐Kustas et al., 2020)
Shark skin	Self-cleaning, underwater drag reduction	(Ball, 1999; Bechert et al., 2000; Bixler and Bhushan, 2013)
Penguin feather	Anti-icing, liquid guidance	(Wang et al., 2016a; Alizadeh-Birjandi et al., 2020)
Butterfly wings	Self-cleaning, liquid-directed	(Qian et al., 1900; Fang et al., 2008)
Spider silk	Collecting water	Zheng et al. (2010)
Earthworm	Drag reduction, lubrication	Zhao et al. (2018)
Mosquito compound eyes	Superhydrophobic, anti-fog	Gao et al. (2007)

The study of superhydrophobic principle can be traced back to 1805, when T. Young (Young, 1805) established Young’s equation of ideal smooth solid surface state, which set a theoretical precedent for studying the wettability of materials. Later, Wensel and Cassie summarized Wensel model (Wenzel, 1936) and Cassie–Baxter model (Cassie and Baxter, 1944) by studying the relationship between surface roughness and wettability. Recent further research shows that superhydrophobic surfaces can be divided into five types (Wang and Jiang, 2007). This classification includes steady-state and transition state, which can explain the phenomena that were difficult to explain by previous theories (Yilgor et al., 2012; Liu et al., 2017a; Chen et al., 2021).

Contact angle and rolling angle are important parameters to characterize the wettability of droplets on solid surfaces, and are also the initial evaluation indexes of biomimetic superhydrophobic surfaces (Aussillous and Quéré, 2001; Richard et al., 2002; Michael and Bhushan, 2007; Nosonovsky and Bhushan, 2008). With the development of research, researchers have made biomimetic superhydrophobic surfaces with multiple functions. Since then, the application fields of biomimetic superhydrophobic surfaces have been expanded by leaps and bounds, included adhesive-responsive superhydrophobic surfaces for sensors (Gao et al., 2019; Liu et al., 2019), industrial anticorrosive superhydrophobic surfaces which can effectively slow down the damage of metal oxide layers and resist strong acid/alkali corrosion (Li et al., 2019c; Ran et al., 2019; Ijaola et al., 2020), superhydrophilic/superhydrophobic surfaces which can realize industrial wastewater treatment and offshore oil spill treatment by using oil–water separation characteristics (Feng et al., 2004; Jayaramulu et al., 2016; Yang et al., 2018; Song et al., 2022), and superhydrophobic coatings with anti-icing and light transmission properties for outdoor glass and photovoltaic converters (Li et al., 2009; Liu et al., 2015a; Cui and Pakkanen, 2020; Zhu et al., 2021). In addition, diversified bionic superhydrophobic surfaces were widely used in modern military (Dong et al., 2013; Wang et al., 2015b; Jiaqiang et al., 2018), microfluidic control (Stratakis et al., 2011; Kong, 2021), fabric and textile industry (Hoefnagels et al., 2007; Xing et al., 2022) and other extended fields.

In this mini review, we reviewed the superhydrophobic surfaces and principles in nature. Section 2 introduced several different preparation methods, with emphasis on the preparation of biomimetic superhydrophobic surfaces by etching modification. Particularly, we discussed the unique advantages and disadvantages of etching modification. Finally, the conclusion of this review and the prospect of the research field in the future were described (Section 3).

## 2 Biomimetic superhydrophobic surface preparation methods

**FIGURE 1:**
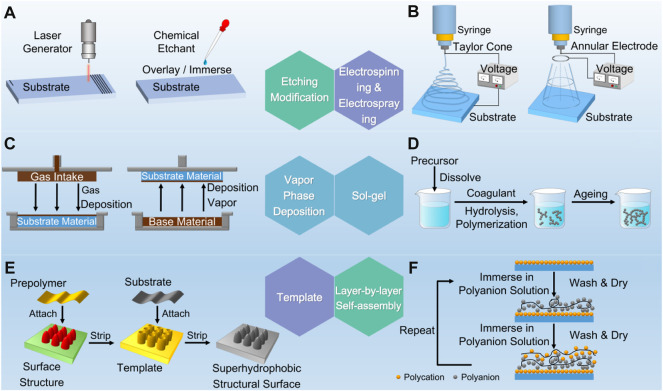
Etching modification **(A)**, electrospinning and electrospraying **(B)**, vapor phase deposition **(C)**, sol–gel **(D)**, template **(E)** and layer-by-layer self-assembly **(F)** methods developed to fabricate superhydrophobic surfaces.

With the deepening of research, the preparation methods of bionic superhydrophobic surfaces were gradually diversified. Figure 1 shows the common preparation methods of superhydrophobic surfaces. Generally speaking, the core idea of preparing biomimetic superhydrophobic surface is to imitate the microstructure of biological surface and modify it with low surface energy substances. According to the order of construction, it can be classified into two categories. The first category is to construct micro-nano rough structures on smooth surfaces, and then decorate them with low surface energy materials. The second category is to directly sketch micro-nano rough structures on low surface energy materials.

In Table 2, we also show the characteristics of typical preparation methods of bionic superhydrophobic materials to understand their advantages and disadvantages. From the table, we can systematically understand the characteristics of various methods, including preparation principle, production cost, production speed, equipment requirement, environmental friendliness, and mechanical durability. It should be emphasized that the characteristics here are for most experiments that use this method to construct bionic superhydrophobic surfaces.

**TABLE 2:** Comparison of preparation methods of superhydrophobic surface.

Preparation method	Principle	Cost	Efficiency	Equipment requirement	Environmental friendliness	Durability	References
Chemical etching	Etchant etching	Inexpensive	Efficient	Low-demand	Harmful	Nondurable	(Liao et al., 2014; Qu et al., 2018; Peng et al., 2019; Attar et al., 2020)
Laser etching	High-energy laser beam	Expensive	Inefficient	High-demand	Harmless	Durable	(Liu et al., 2017b; Pan et al., 2019; Yang et al., 2019; Zheng et al., 2020; Li et al., 2021d)
Chemical vapor deposition	Chemical vapor reaction	Inexpensive	Fair	Low-demand	Harmful	Durable	(Jiang et al., 2016; Sun et al., 2017; Aljumaily et al., 2018; Guo et al., 2019)
Physical vapor deposition	Vaporization followed by deposition	Inexpensive	Efficient	High-demand	Harmless	Fair	(Du et al., 2020; Böke et al., 2016; Tavana et al., 2006)
Electrospinning and electrospraying method	Droplet spraying and stretching in electric field	Inexpensive	Efficient	Low-demand	Harmless	Nondurable	(Ke et al., 2014; Du et al., 2021; Deo et al., 2022)
Sol-gel method	Hydrolytic condensation of compounds under liquid phase	Inexpensive	Inefficient	Low-demand	Harmful	Nondurable	(Tadanaga et al., 2000; Manoharan et al., 2021)
Template method	Post-compression modifications	Fair	Inefficient	Low-demand	Harmless	Durable	(Lee et al., 2010; Xu et al., 2011; Zhang et al., 2020a)
Layer-by-layer self-assembly method	Inter-particle electrostatic interaction	Inexpensive	Inefficient	Low-demand	Harmless	Nondurable	(Yang et al., 2013; Brown and Bhushan, 2015)

Among these methods, the etching modification method will be described in detail later. In addition, it is not difficult to find that the above-mentioned methods all have a common feature, namely, self-cleaning. At present, self-cleaning has basically become a common property of superhydrophobic surfaces (Parkin and Palgrave, 2005; Sethi and Manik, 2018). The most direct way to achieve self-cleaning effect is to use extremely low rolling angle. The accumulated dirt particles can be effectively cleaned by fast-sliding water droplets (Liu et al., 2015b; Sharma et al., 2021). Photocatalytic reaction can effectively decompose pollutants and achieve the purpose of more efficient self-cleaning (Afzal et al., 2014; Wang et al., 2021a). Compared with simply using droplets to roll away dirt, this more active self-cleaning method is more suitable for places with more pollutants such as oil pollution and organic matter (Mor et al., 2004; Rus et al., 2013; Moghaddasi and Mohammadizadeh, 2022).

In modern research, before the formulation and product development of self-cleaning superhydrophobic materials, molecular dynamics is more and more used for simulation to predict the target performance, which can take into account the self-cleaning performance and the durability brought by the adhesion between film and substrate (Sethi et al., 2022). Through molecular dynamics simulation optimization and experimental verification, Sethi’s research team comprehensively predicted and explained the surface behavior, substrate adhesion, and overall performance of the blend, which was beneficial to determine the volume, surface, and interface characteristics of the best formula before the preparation of bionic superhydrophobic surface coating, reducing the workload in actual preparation, improving work efficiency and product performance (Sethi et al., 2019; Sethi et al., 2020a; Sethi et al., 2020b).

### 2.1 Etching modification methods

Etching is a simple and effective method to achieve bionic superhydrophobic effect. This method is through selective etching to realize the processing of micro-nano double-ordered structure on the substrate surface (Ellinas et al., 2021).

Wet etching and dry etching are two main ways of etching. In wet etching, the etching substrate is soaked or coated with chemical reagents (such as acid, alkali, etc.), and the etching solution reacts with the material to remove specific surface materials (Huang et al., 2011; Yeganeh and Mohammadi, 2018; Jayarama et al., 2021). Dry etching is a process that uses laser or plasma to react with the substrate surface to form volatile substances, or directly bombards the substrate surface to corrode it (Lee, 1979; Elhadj et al., 20122012; Wang et al., 2021b; Fan et al., 2021). The following section will introduce wet chemical etching and dry laser etching, as well as their application scope, advantages and disadvantages.

#### 2.1.1 Chemical etching method

**FIGURE 2:**
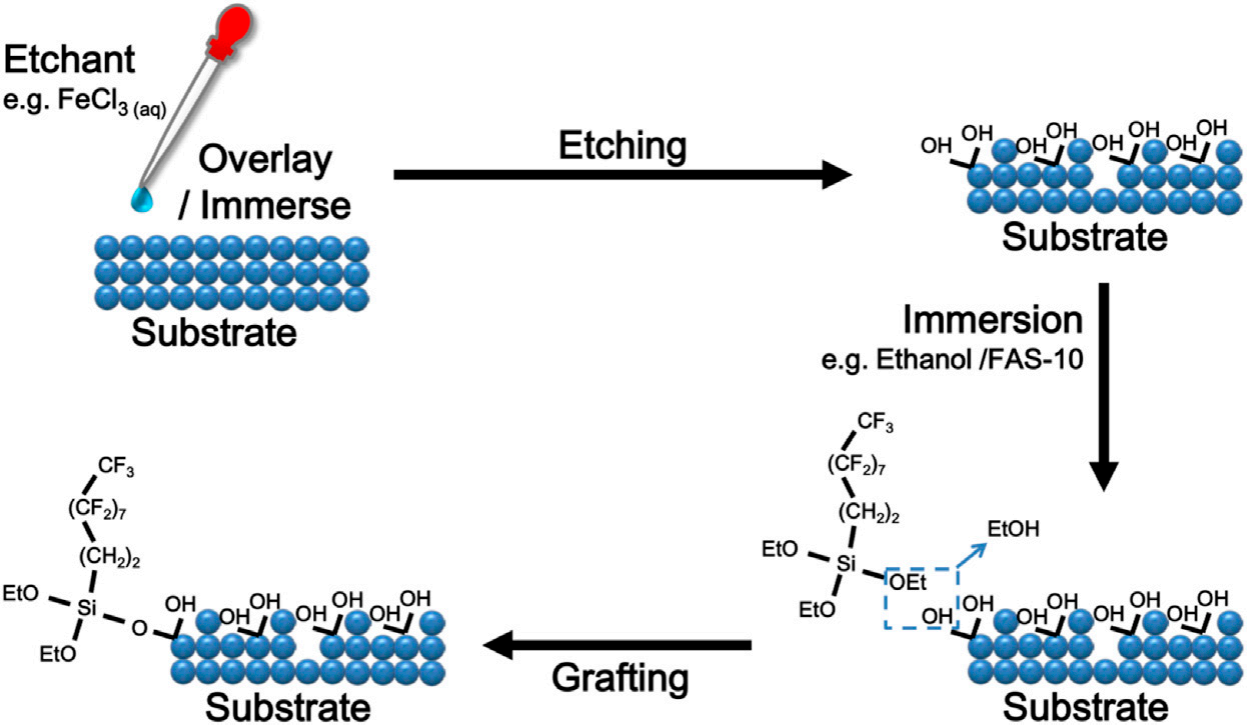
Schematic diagram of chemical etching and grafting.

Generally, metals and alloys are the most suitable substrates for chemical etching, especially magnesium alloys or aluminum alloys (Panda, 2021; Peng et al., 2021). By controlling the concentration of etching solution and etching time, the structural characteristics of superhydrophobic surface, such as roughness, can be effectively changed (Wei et al., 2021b; Guo et al., 2021; Krishnan et al., 2021; Zheng et al., 2021) (Figure 2).

Strong acids, such as HCl and H_2_SO_4_ are common and effective etching solutions. Kumar et al. (Kumar and Gogoi, 2018) used HNO_3_-HCl mixture to etch aluminum plate, and then treated it with high-density hexadecyl trimethoxysilane (HDTMS) toluene solution to construct a superhydrophobic surface with the static contact angle of 162.0 ± 4.2° and the rolling angle of 4 ± 0.5°. Besides good superhydrophobic self-cleaning performance, the surface also showed good thermal stability, chemical stability, and mechanical stability. Nguyen et al. (Nguyen et al., 2021) etched with HCl solution and deposited with FOTS (fluorooctatrichlorosilane) to prepare superhydrophobic aluminum plates with low ice adhesion strength and long freezing time. In addition, a model for calculating the freezing time was put forward, and the comparison between the experimental and theoretical calculation showed excellent consistency, which provided guidance for understanding the icing phenomenon and designing the ice-repellent surface. Campbell et al. (Gray-Munro and Campbell, 2017) combined the chemical etching of H_2_SO_4_-H_2_O_2_ with the deposition of silicone coating to construct a lotus leaf-like structure on the surface of magnesium alloy, which had excellent stability in aqueous solution. By this way, the surface degradation rate of biodegradable superhydrophobic magnesium alloy with biocompatibility was controlled, which was significant in medical application. Saleh et al. (Saleh and Baig, 2019) functionalized stainless steel with octadecyl trichlorosilane (ODTCS) after structural treatment with H_2_SO_4_, and obtained superhydrophobic and superhydrophilic properties. Experiments showed that the separation efficiency of various nonpolar organic components from water was over 99%, which was expected to be used to separate oil from water. In addition, HF in inorganic acid is also a good etching solution (Qu et al., 2007; Zhang et al., 2015; Du et al., 2018; Kim et al., 2018; Xu et al., 2019; Zhang et al., 2020b; Shaikh et al., 2021).

Compared with inorganic acids, organic acids are less used for etching, because most inorganic acids are relatively more stable and lower in cost. Ou et al. (Ou et al., 2019) sprayed cold galvanized coating on iron substrate, then etched with acetic acid, and finally modified with stearic acid. The superhydrophobic sample surface with an apparent contact angle of 168.4 ± 1.5° and a rolling angle of 3.5 ± 1.2° was obtained. This sample combined the respective characteristics of zinc coating and superhydrophobic surface in terms of metal corrosion resistance, which had rapid manufacturing process, good mechanical durability, and easy repair. Wu et al. (Wu et al., 2012) compared the etching effects of aluminum alloy samples in three different acid solutions (acetic acid, hydrochloric acid, and oxalic acid), and found that the mixed solution of oxalic acid and hydrochloric acid was the better etching combination. Similarly, they found that better surface roughness can be obtained by adjusting the concentration of Cl^−^ ion and oxalate ion in acid solution. This provided a new strategy for controllable preparation of superhydrophobic films on aluminum alloys, which could be used in practical industrial applications. Chen et al. (Chen et al., 2010) proposed a superhydrophobic surface preparation method without hydrophobic treatment, which was directly obtained by soaking aluminum in the mixed solution of HCl and acetic acid. In addition, they also studied the effect of acetic acid content on the surface structure.

Besides acidic solutions, other kinds of solutions have been widely used. NaOH is the most commonly used alkaline etchant, which is usually used to etch aluminum alloy (Saleema et al., 2010; Huang et al., 2015; Lomga et al., 2017; Nguyen-Tri et al., 2019a; Tudu et al., 2019; Yang et al., 2022). In Peng’s work (Peng and Deng, 2015), NH_3_ was selected as the etchant of aluminum, and the superamphiphobic sample obtained by hot ammonia solution etching and fluorosilane modification had excellent chemical stability. This simple, economical, environment-friendly, and efficient method could be used in the fields of oil-proof and water-proof. Wan et al. (Wan et al., 2018) combined ammonia etching and hydrothermal treatment to construct superhydrophobic surface on copper substrate modified by 1H,1H,2H,2H-perfluorodecyl triethoxysilane (PFDTES), which showed good waterproof, anti-corrosion and anti-adhesion properties in simulated seawater and humid air. Parin et al. (Parin et al., 2018) used three different salt solution etchants, namely AlCl_3_, FeCl_3_ and CuCl_2_, to obtain superhydrophobic aluminum surfaces by chemical etching and fluorosilane deposition, respectively, which confirmed that different etchants would produce different surface micro-nanostructures. Rodič et al. (Rodič et al., 2020) etched aluminum in FeCl_3_ solution, and then grafted at ambient temperature directly in an ethanol solution of 1H,1H,2H,2H-perfluorodecyltriethoxysilane. The prepared superhydrophobic surface has the characteristics of self-cleaning and anti-icing. Moreover, the promotion of dropwise condensation significantly improved the heat transfer coefficient, so that the sample could be widely used in heat transfer industry. Wang et al. (Wang et al., 2016b) chemically etched magnesium in CuCl_2_ solution, and then modified it with oleic acid to prepare superhydrophobic surface with contact angle of 155°. Song et al. (Song et al., 2012) developed a rapid preparation method of superhydrophobic materials, in which aluminum was immersed in CuCl_2_ solution and then modified with ethanol solution of fluoroalkyl silane, and the whole process only took a few seconds. This convenient and efficient method may have the potential of large-scale preparation.

Chemical etching can be divided into two-step method (Milosev et al., 2017; Milošev et al., 2019; Rodič et al., 2022) and one-step method (Chen et al., 2010) in addition to the classification of etching solution. This is classified according to the operation steps. The two-step method is chemical etching and then coating, while the one-step method is chemical etching and coating in the same step (Varshney et al., 2016).

Chemical etching is a simple, quick and low-cost method, which can be used in a large scale. However, the etching solution used in the operation process is toxic to human health and the environment. In addition, the specific shape and thickness of the micro-nano double-ordered structure are difficult to control, and the mechanical durability of the surface is not high. Under the condition of over-etching, the microsurface roughness of the substrate decreases, and even the basic mechanical properties of the substrate are destroyed, which is also an important problem. Therefore, the preparation of green etchant, the optimization of etching process, the precise control of substrate surface morphology and thickness, and the reduction of environmental pollution risk are the key development paths that chemical etching needs to explore in the future.

#### 2.1.2 Laser-etching method

**FIGURE 3:**
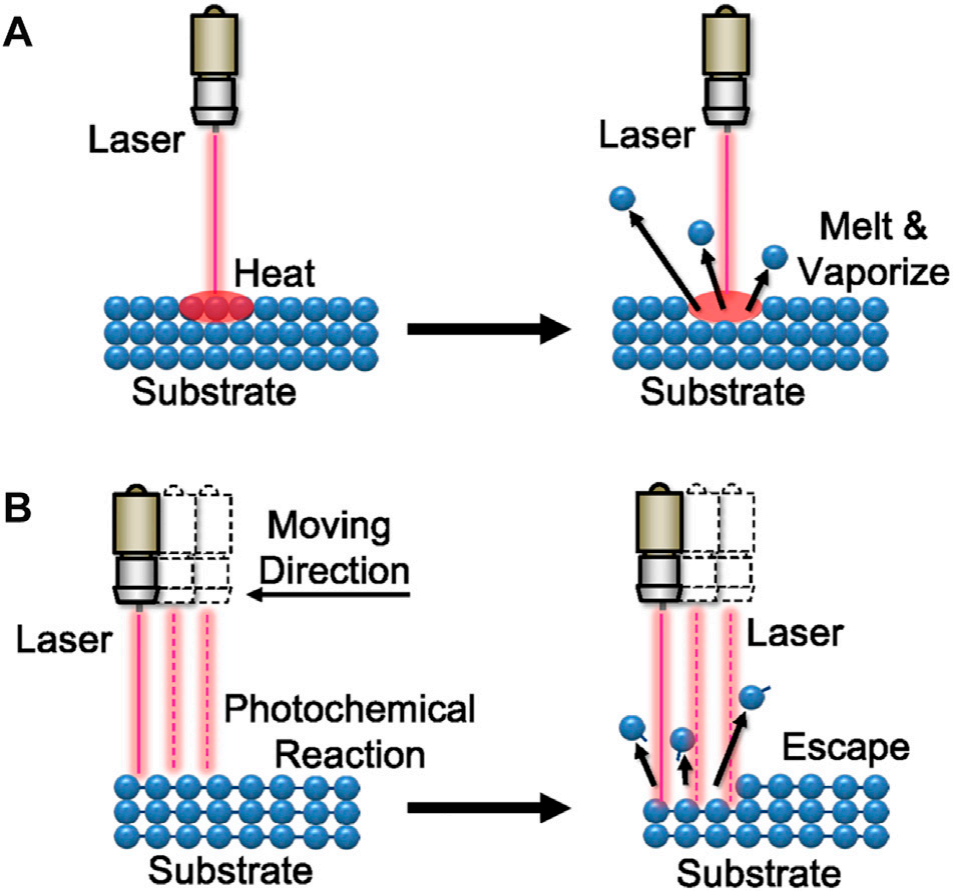
Schematic diagram of **(A)** laser heat treatment and **(B)** cold treatment.

As shown in Figure 3, laser etching can be divided into heat treatment and cold treatment according to its reaction principle (He et al., 2019; Ehrhardt et al., 2021). Irradiating the substrate surface with high-energy laser beam, the generated high temperature melts and vaporizes the material in a short time. After cooling, the superhydrophobic surface is constructed, which is called laser heat treatment. Cold treatment is a method of breaking chemical bonds in materials through photochemical reaction and constructing superhydrophobic surfaces after cooling. This technology has been widely used in various materials, including metal, glass, and polymer (Fadeeva et al., 2011; Lu et al., 2017; Dinh et al., 2018; Kostal et al., 2018; Li et al., 2019d; Ma et al., 2019; Stratakis et al., 2020).

Boinovich et al. (Boinovich et al., 2017) used Al–Mg alloy as the substrate, and proved that laser treatment can endow the surface with multi-peak roughness and change the composition of the surface layer. This research had overcome the obstacles of metal materials in industrial application, such as easy corrosion, poor cold resistance or weak thermal shock stress resistance. Li et al. (Li et al., 2018) prepared periodic microscale papillary pit microstructure on magnesium alloy surface by laser ablation. After chemical etching with AgNO_3_ and surface modification with stearic acid, the prepared surface has superhydrophobic property, and the maximum contact angle reaches 158.2°. By adjusting the microstructure, such as the center distance of pits, the surface can change from low adhesion to high adhesion. In addition, due to the high degree of independent selection and accuracy of laser etching, the surface structure that meets the requirements of experimenters can be perfectly reproduced. Using this characteristic of laser etching, superhydrophobic surfaces with excellent anisotropy can be easily prepared (Yong et al., 2014; Fang et al., 2018; Lasagni et al., 2018; Cai et al., 2019; Tuo et al., 2019; Bai et al., 2020; Rong et al., 2021; Yang et al., 2021). In another study, femtosecond laser and picosecond laser were used to construct nanostructures of aluminum, copper, and galvanized steel, respectively, and then aging in vacuum was used to replace low surface energy modification. This method of combining ultrafast laser surface nanostructures with vacuum aging was suitable for a wide range of self-cleaning applications (Khan et al., 2021). In addition to the metal matrix, Chen et al. (Chen et al., 2018) showed a method of manufacturing bionic reed leaves by laser treating the surface of structured polydimethylsiloxane (PDMS). Du et al. (Du et al., 2022) proposed a one-step laser-etching method for manufacturing superhydrophobic silicone rubber with bionic layered micro/nano structure, whose contact angle and sliding angle can be adjusted according to the number of laser-etching cycles, which is beneficial to different application requirements. It was a potential candidate to protect flexible electronics equipment in rainy days and acid/alkali environments. Inspired by the hexagonal microstructure array of mosquito’s compound eye, He et al. (He et al., 2021) made multifunctional superhydrophobic self-cleaning glass with anti-fog and anti-icing by laser texturing. Jing et al. (Jing et al., 2022) used picosecond laser to etch glass substrate and chemically modified it by silanization to prepare superhydrophobic surface with high adhesion. They also pointed out that laser-induced micro/nano structure depends on laser energy to a great extent and significantly affects adhesion, while scanning times have a slight effect on surface morphology and adhesion.

**FIGURE 4:**
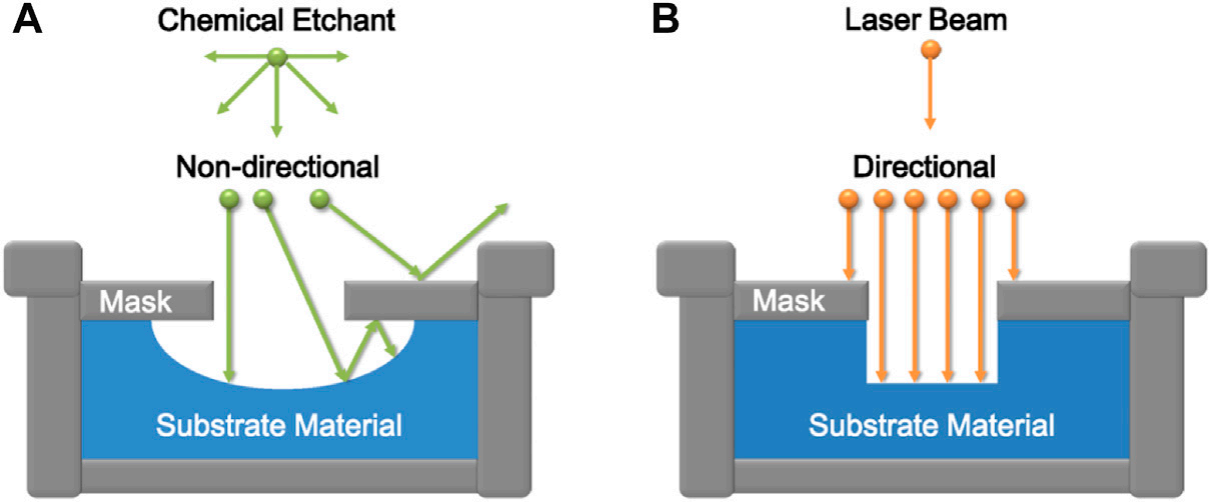
Directional comparison between **(A)** chemical etching and **(B)** laser etching.

Laser-etching method has high precision and controllability. By controlling the laser type, irradiation time and light intensity, the surface microstructure with controllable direction can be obtained on different substrates (Figure 4), which is suitable for most materials. In addition, no harmful substances are produced during the experiment, which is undoubtedly environment-friendly. In recent years, with the rapid development of 3D laser printing technology and femtosecond laser technology, researchers have been able to complete custom etching of various complex microstructures. However, because the laser synthesis equipment is very complex, expensive, energy-intensive, and the action area of a single laser beam is relatively small, it is not suitable for industrial large-scale preparation of superhydrophobic materials, and it is currently in the laboratory stage.

#### 2.1.3 Mixed-etching method

The etching methods mentioned in the above two chapters usually use different substrates (Table 3), but they can actually be used in combination. For example, Xia et al. (Xia et al., 2022) compared three methods: chemical etching, laser etching, and chemical-laser mixed etching, and pointed out that specific chemical-laser mixed etching parameters can effectively prepare uniform hierarchical structure on aluminum alloy surface, achieving excellent hydrophobicity, and low ice adhesion. When this method was applied to the wing manufacturing, the obtained wing may prevent the aircraft from freezing and protect the aerospace safety. Dong et al. (Dong et al., 2011) also prepared a hydrophobic copper surface with tunable regular microstructure and random nanostructure whose water contact angle is about 153°. The regular microstructure was obtained by nanosecond pulse laser etching, while the random nanostructure was determined by chemical etching. The regular microstructure was obtained by nanosecond pulse laser etching, while the random nanostructure was determined by chemical etching. Liu et al. (Liu et al., 2013) dipped the aluminum alloy in HNO_3_ and Cu(NO_3_)_2_ after laser processing, and finally modified it with DTS (CH_3_(CH_2_)_11_Si(OCH_3_)_3_) to prepare the bionic superhydrophobic surface with high adhesion. It is easy to find that these two methods can also be used together with other etching methods to achieve better bionic superhydrophobic effect (Li et al., 2014; Gu et al., 2017; Tian et al., 2017; Feng et al., 2022a; Feng et al., 2022b). Rodič et al. (Rodič and Milošev, 2019) made highly hydrophobic aluminum surface in NaOH solution containing various alkoxysilanes by one-step ultrasonic process, which had corrosion resistance, self-cleaning, and anti-icing characteristics. These mixed preparation processes provided a new way for the preparation of various materials.

**TABLE 3:** Simple summary of common etching methods of different substrates.

Substrate	Common methods	References
Aluminum (Al)	Chemical Etching (Cl^−^ Ion)	(Ran et al., 2019; Zhang et al., 2019; Ellinas et al., 2021)
Magnesium (Mg)	Chemical Etching (SO_4_^−^ Ion)	(Ran et al., 2019; Ellinas et al., 2021; Peng et al., 2021)
Stainless Steel	Chemical Etching (Cl^−^ Ion)	(Nanda et al., 2019; Saleh and Baig, 2019; Ellinas et al., 2021)
Glass	Laser Etching (Plasma)	(Li et al., 2018; Wu et al., 2021; Gao et al., 2022)
Polymer	Laser Etching (Plasma)	(Dimitrakellis and Gogolides, 2018; Lee et al., 2018; Nguyen-Tri et al., 2019b; Nageswaran et al., 2019)

## 3 Summary

This article summarized the basic principle and model of superhydrophobic, epitomized the structure and wetting characteristics of biological superhydrophobic surfaces in nature, reviewed various preparation methods of bionic superhydrophobic materials, emphasized the etching method, and discussed the research status and challenges of superhydrophobic applications. As described in detail above, the chemical etching method is simple, fast, low-cost and has the potential for large-scale application. Its disadvantage is that it is harmful to the environment and human body, and excessive etching will cause the mechanical properties of samples to be destroyed. Laser etching has controllable and customizable surface morphology, but it is expensive and inefficient. The mixed use of the two methods has indeed achieved the effect of complementing each other’s strengths to a certain extent, but it is undeniable that the equipment price and pollution have not been improved.

## 4 Outlook

Science and technology originates from nature and is superior to nature. The research of bionic superhydrophobic surface started from the early imitation of natural animal and plant surface structures, and now it has expanded to the creative behavior of designing structures and modifying materials independently, especially the birth of laser etching, which greatly improved the structural accuracy. The related properties of superhydrophobic surfaces have also developed from simple hydrophobicity to many directions, including anti-icing, water collection, directional transportation and wetting behavior transformation, which has brought about significant changes in the fields of industrial life and national defense science and technology development. The single bionic superhydrophobic surface can no longer meet the actual needs, and the research of multifunctional bionic superhydrophobic surface has become a hot topic discussed by scholars. However, with the continuous expansion and deepening of the research in the field of etching, the existing problems in the process of preparing bionic superhydrophobic surfaces by etching are also exposed. For example, the environmental friendliness and mechanical properties of chemical etching need to be improved, and laser etching needs to consider how to improve efficiency, reduce equipment costs and achieve mass production. In view of the research hotspots and existing problems, we put forward the following prospects:

(1) Superhydrophobic surface should be further developed toward multifunction. Based on the superhydrophobic function, the surface has many functions, such as antibacterial (Zhan et al., 2022), anti-ultraviolet, anti-radiation, underwater drag reduction, and performance change. It improves the applicability of superhydrophobic materials in many fields and environments, and has far-reaching significance for industrial application. Additionally, if the micro-nano structure is directly built on stealth coating materials (wave absorbing materials, light transmitting materials, light guiding materials, etc.), it will have the characteristics of anti-reflection, anti-radiation, and drag reduction, further increasing its concealment and maneuverability. The stealth surface made by this method is likely to make a major breakthrough in modern national defense and military fields.
(2) To improve the chemical and mechanical stability, the appearance of self-healing superhydrophobic coating provides a new development direction for related research (Sam et al., 2019; Chang et al., 2020). The mechanism of intrinsic self-healing superhydrophobic coating is to introduce dynamic chemical bonds into the internal structure of the material. When the material is damaged, the damaged chemical bonds will be restored to the initial state due to dynamic equilibrium, so that the structure and state of the material can be restored. The external self-repairing super-hydrophobic coating can be stimulated by changing the conditions, such as light and temperature, so that the repairing agent inside the material can be released and migrated to the damaged part, and thus the damaged surface can be healed. However, considering the complex environment in practical engineering application, besides self-healing, the wear resistance, acid and alkali resistance and long-term weather resistance of superhydrophobic coating itself need to be further improved.
(3) For the materials with weak hydrophobicity and easy to be polluted by oily substances, it is necessary to further study the super-double hydrophobic materials that are both hydrophobic and oleophobic, and make them into responsive materials, that is, to switch or switch the surface free energy by external stimulation. In addition, perfluoro silanes, which has both hydrophobic and oleophobic properties, is a target worthy of consideration.
(4) Aiming at the problems of strong pollution and high-energy consumption, the development of new biomaterials and new energy sources provides some guiding ideas for the preparation of green, environmentally friendly, low-cost, and reliable superhydrophobic surfaces.
(5) The effects of surface geometry size, wettability, and surface composition on superhydrophobic properties, especially the quantitative research directly related to hysteresis angle, need to be deepened, which is not only to be studied by etching method, but also to be explored by all methods.
(6) It is still necessary to study the preparation of superhydrophobic materials at low cost to enhance the practicability and expand the application fields.

To sum up, the future development direction of preparing bionic superhydrophobic surface by etching method is to combine the advantages of the two methods and develop a set of durable, energy-saving, low-cost, and mass-production preparation methods that meet the multifunctional application requirements of superhydrophobic surface. Only by realizing the industrial production of superhydrophobic surfaces can we really get out of the laboratory and into life.
